# Liver cyst with biliary communication treated with endoscopic ultrasound-guided drainage: A case report

**DOI:** 10.1097/MD.0000000000029007

**Published:** 2022-03-18

**Authors:** Kento Shionoya, Kazuya Koizumi, Sakue Masuda, Yuma Suno, Jun Kawachi, Karen Kimura, Makomo Makazu, Jun Kubota, Takashi Nishino, Chihiro Sumida, Junichi Tasaki, Chikamasa Ichita, Akiko Sasaki, Hiroki Hadano, Makoto Kako

**Affiliations:** ^a^ *Shonan Gastroenterology Medicine Center, Shonan Kamakura General Hospital, Okamoto 1370-1, Kamakura, Kanagawa, Japan,* ^b^ *Department of General Surgery, Shonan Kamakura General Hospital, Okamoto 1370-1, Kamakura, Kanagawa, Japan,* ^c^ *Department of Emergency, Shonan Kamakura General Hospital, Okamoto 1370-1, Kamakura, Kanagawa, Japan.*

**Keywords:** endoscopic ultrasound-guided cyst drainage, liver cyst with biliary communication, percutaneous transhepatic cyst drainage

## Abstract

**Rationale:**

Simple liver cysts are common, and usually benign and asymptomatic, requiring little to no treatment. Liver cysts with biliary communication, however, are rare and require effective treatment to avoid recurrence.

**Patient concerns:**

A 70-year-old woman with breast cancer visited our hospital for treatment. Physical examination revealed abdominal distension and bilateral lower leg edema.

**Diagnosis:**

Abdominal contrast-enhanced computed tomography revealed a giant liver cyst, inducing inferior vena cava compression that was causing her edema.

**Interventions:**

Percutaneous transhepatic cyst drainage was performed. Since the bilirubin level in the drained fluid was high, the patient was diagnosed with a liver cyst with biliary communication. After the procedure, her symptoms improved and the cyst decreased in size. However, the drainage volume did not decrease after approximately 2 weeks. Sclerotherapy with minocycline was ineffective. Thus, endoscopic retrograde cholangiopancreatography was performed, and an endoscopic nasobiliary drainage tube was inserted. The percutaneous drainage tube was clamped, and the cyst showed increase in size. Therefore, endoscopic ultrasound-guided cyst drainage, which is less invasive than surgery, was performed.

**Outcomes:**

The cyst tended to decrease in size even after the percutaneous drainage tube had been removed. At 3years follow-up, the cyst has almost disappeared.

**Lessons:**

Endoscopic ultrasound-guided drainage can treat liver cyst with biliary communication.

## 1. Introduction

Simple liver cysts are common, usually asymptomatic, and can be detected incidentally on abdominal imaging. However, liver cysts with biliary communication are rare, and in some cases, patients are symptomatic. Trauma, percutaneous drainage, or infection of the cysts can cause communication between the liver cysts and the bile duct. Symptomatic treatment is necessary for abdominal pain, jaundice, and other complications of infection. The most commonly performed interventions are percutaneous drainage, sclerotherapy with minocycline, and endoscopic transduodenal papillary drainage. Surgical treatment may be considered for cases that fail to improve after initial intervention.^[1-3]^ We report a case of a liver cyst with biliary communication, treated via endoscopic ultrasound (EUS)-guided cyst drainage.

## 2. Case presentation

A 70-year-old woman with breast cancer visited our hospital for treatment. Physical examination revealed abdominal distension and bilateral lower leg edema. Laboratory tests showed elevated hepatobiliary enzyme levels (aspartate aminotransferase, 61 U/L; alanine transaminase, 57 U/L; lactate dehydrogenase, 733 U/L; alkaline phosphatase, 2713 U/L; gamma-glutamyl transpeptidase, 736 U/L; total bilirubin, 3.0 mg/dL; direct bilirubin 2.1 mg/dL), a normal white blood cell count of 5200/mm^3^, high C-reactive protein level of 1.08 mg/dL, and a low albumin level of 2.3 mg/dL. Abdominal contrast-enhanced computed tomography (CT) revealed a large cyst in the liver and significant ascites fluid accumulation (Fig. 1). Giant liver cyst caused compression of the inferior vena cava, resulting in ascites and bilateral leg edema. The patient underwent percutaneous transhepatic cyst drainage for the giant liver cyst (Fig. 2). The bilirubin level in the puncture fluid was high, based on which she was diagnosed as liver cyst with biliary communication. After percutaneous drainage, the CT revealed that the size of the cyst had decreased (Fig. 3). Her symptoms also improved, but the amount of drainage did not decrease after approximately 2 weeks. Therefore, sclerotherapy with minocycline was performed repeatedly. However, the drainage amount still did not decrease. Endoscopic retrograde cholangiopancreatography (ERCP) showed communication between the liver cyst and bile duct. A guidewire was inserted into the cyst, but ERCP was unsuccessful. An endoscopic nasobiliary drainage tube was then inserted to drain the common bile duct (Fig. 3). The percutaneous drainage tube was clamped, but the cyst showed increase in size (Fig. 4). Although surgery was considered, the typical cyst deroofing procedure was deemed insufficient due to the communication between the cyst and bile duct. In cases wherein the communication is undetectable, hepatic resection is necessary. In this case, early initiation of breast cancer treatment was preferred; therefore, EUS-guided cyst drainage, which is less invasive than surgery, was considered. EUS (UCT-Q260; Olympus, Tokyo, Japan) was used for the drainage. The liver cyst was punctured using a 19-gauge needle (SonoTip, Medico’s Hirata Inc. Osaka, Japan) via the gastric wall, and a guidewire (VisiGlide 2, Olympus, Tokyo, Japan) was inserted into the cyst. The needle tract was then dilated using a mechanical dilator (7 Fr ES Dilator, Zeonmedical, Tokyo, Japan), and a 7 Fr × 15cm double-pigtail plastic stent was inserted (Fig. 5). The patient responded favorably to the endoscopic treatment, with the cyst size decreasing even after the removal of the percutaneous drainage tube. After 3 years of endoscopic treatment, the cyst has almost completely shrunk according to CT imaging (Fig. 6).

**Figure:**
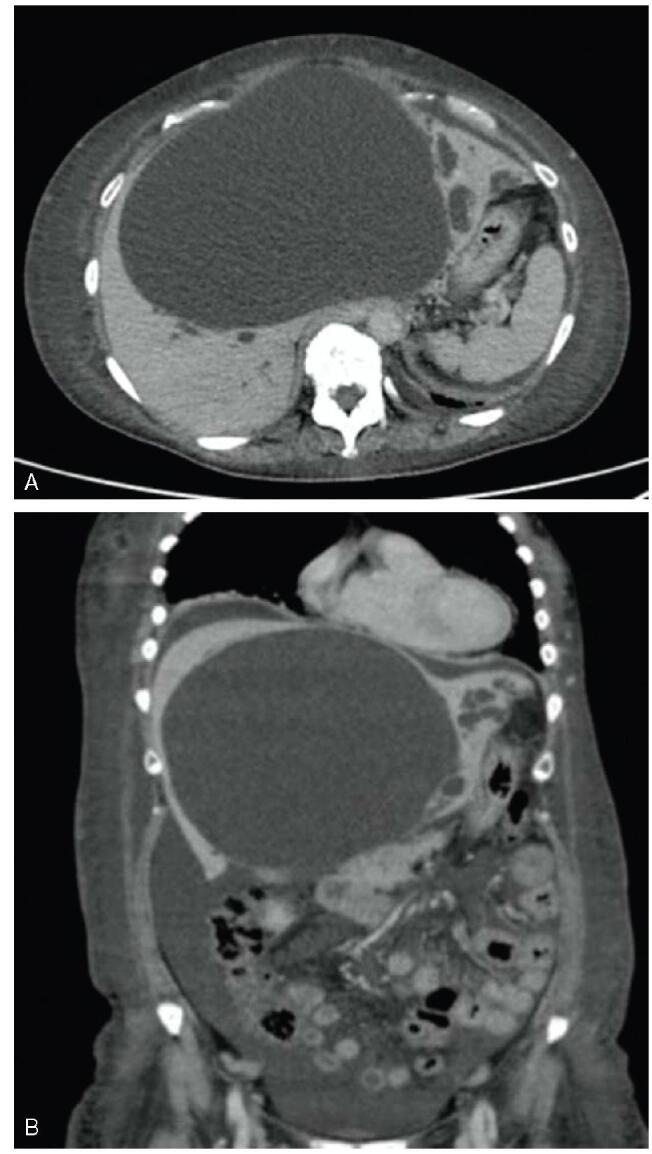
**Figure 1.** Abdominal contrast-enhanced computed tomography scans obtained during hospitalization. A) A large cyst is visible in the liver. Dilation of the intrahepatic bile ducts are also observed. B) Presence of significant ascites.

**Figure:**
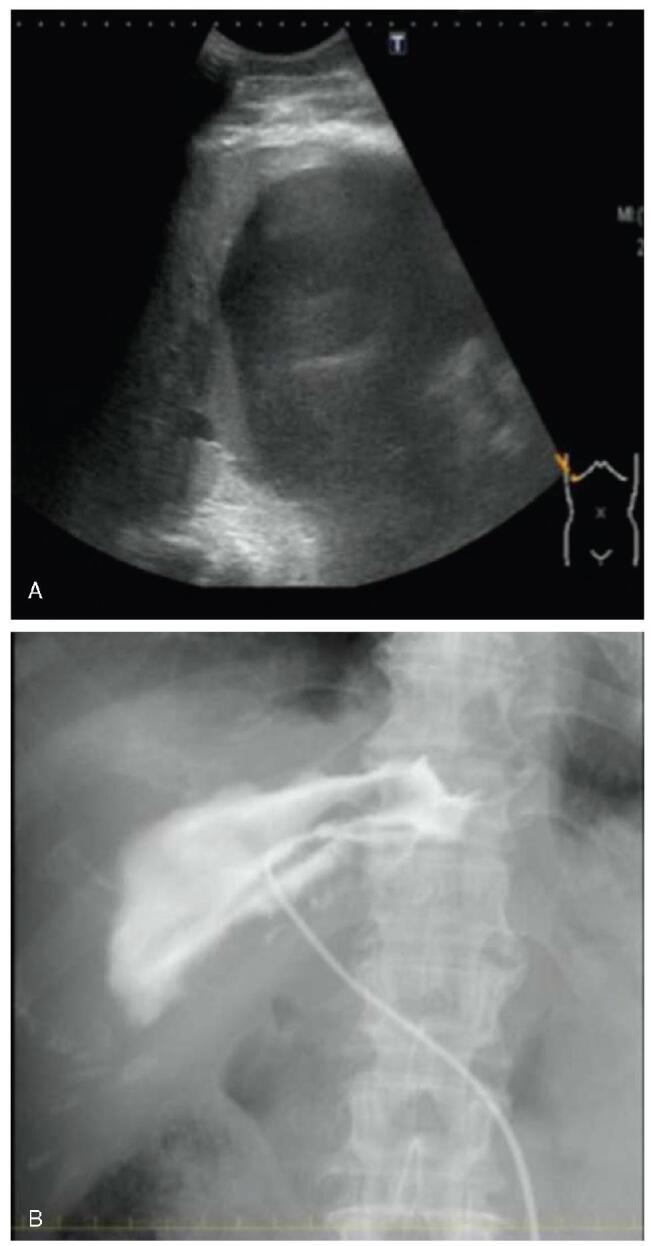
**Figure 2.** Ultrasonography-guided percutaneous transhepatic cyst drainage was performed for the giant liver cyst. A) Ultrasound showing the liver cyst. B) Fluoroscopic image showing the stent placed in the cyst.

**Figure:**
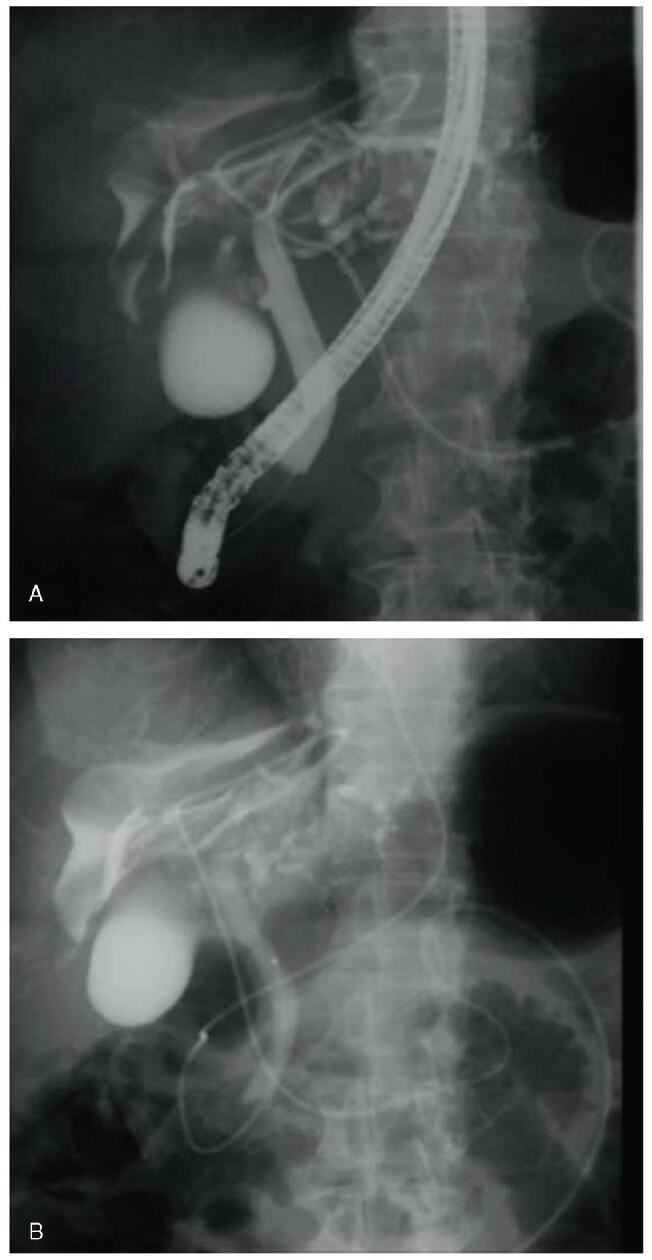
**Figure 3.** Endoscopic retrograde cholangiopancreatography was performed for additional biliary drainage. Cholangiography showing contrast leakage from the bile duct into the cystic cavity. It was challenging to insert the guidewire into the cyst; therefore, we placed an endoscopic nasobiliary drainage tube in the common bile duct.

**Figure:**
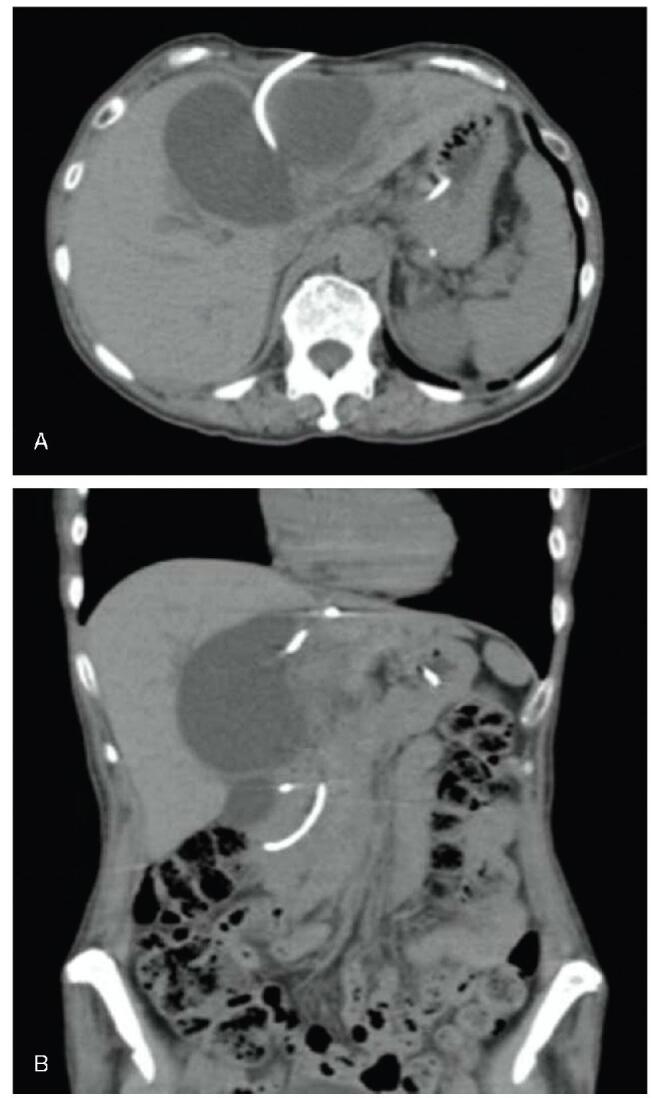
**Figure 4.** Abdominal computed tomography (CT) showing enlargement of the liver cyst. After the endoscopic nasobiliary drainage tube was placed in the common bile duct, and the percutaneous drainage tube was clamped, abdominal CT was performed, which showed that the liver cyst increased in size.

**Figure:**
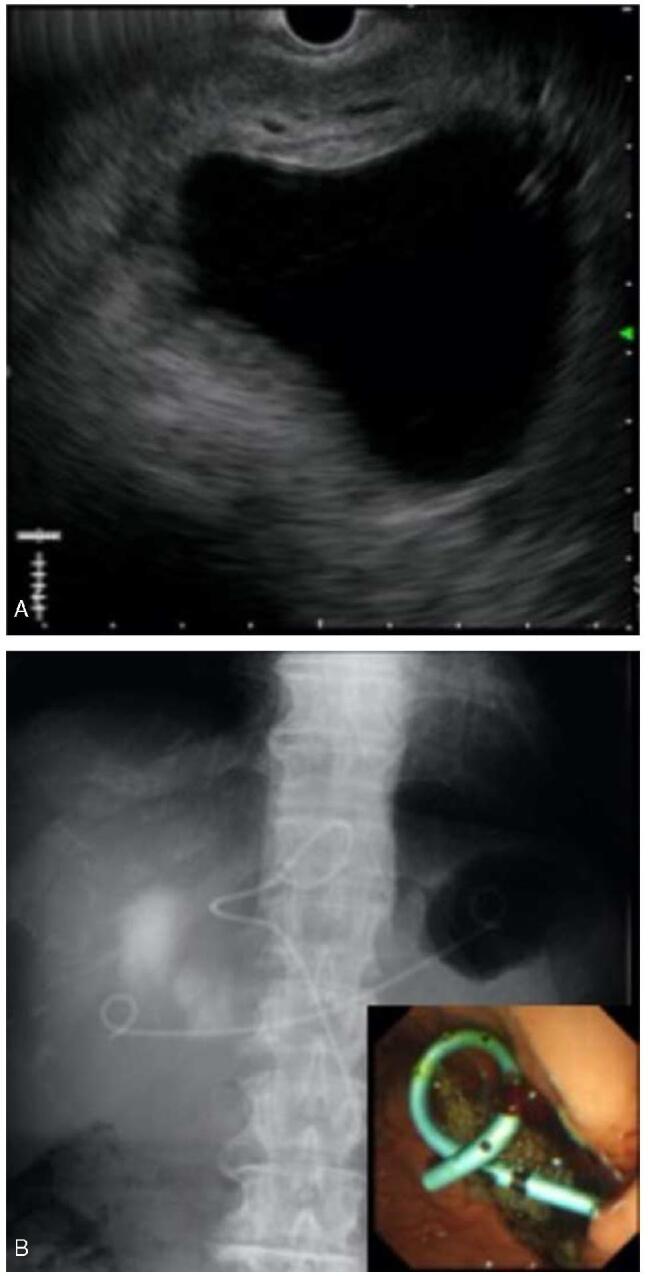
**Figure 5.** Endoscopic ultrasound-guided drainage was performed for the liver cyst with biliary communication. A) Endoscopic ultrasound showing a liver cyst. B) A 7 Fr × 15 cm double pigtail plastic stent was placed in the liver cyst.

**Figure:**
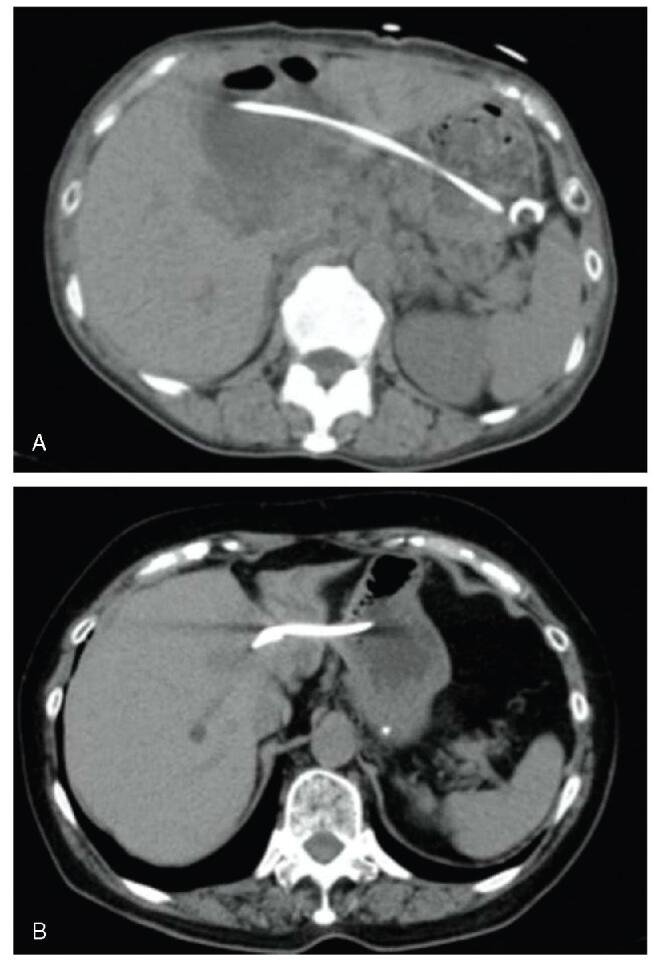
**Figure 6.** Abdominal plain computed tomography (CT) scans obtained after endoscopic ultrasound-guided drainage. A) CT imaging performed 4days after the procedure showing that the size of the liver cyst was decreased. B) CT imaging performed 3years after the procedure showing that the cyst has almost disappeared.

## 3. Discussion

Simple liver cysts are typically detected as an incidental finding on abdominal imaging. Most of these cysts are benign, present asymptomatically, and require no treatment. For symptomatic patients, various treatments, including percutaneous drainage, cystojejunostomy, deroofing or excision of the cyst, partial liver resection, and endoscopic drainage are indicated. Although percutaneous drainage is the most common treatment for liver cysts,^[4-7]^ it has been associated with a high recurrence rate.^[8,9]^ Sclerotherapy with ethanol or minocycline hydrochloride is indicated for cases that do not improve with percutaneous drainage. However, the recurrence rate of liver cysts following percutaneous drainage is high, regardless of additional sclerotherapy, and some patients eventually require surgical treatment.^[1,8]^

ERCP has been reported to be an effective treatment option for liver cysts with biliary communication that were unresponsive to percutaneous drainage.^[10]^ In our case, ERCP and endoscopic nasobiliary drainage tube placement was performed, but proved ineffective. Surgical treatment was then performed. Deroofing of the cyst is the standard treatment for cases of solitary cysts or polycystic liver diseases without malignancy or biliary communication because of its simplicity, effectiveness, and low morbidity rate.^[11-13]^ Previous reports have discussed the usefulness of laparoscopic deroofing for liver cysts with biliary communication. However, it proved to be inappropriate for cases with biliary communication because the bile ducts would need to be treated to prevent postoperative bile leakage, with treatment of the bile ducts possibly leading to hepatic resection.^[2,14,15]^ In this case, since early breast cancer treatment was preferred, minimally invasive EUS-guided drainage was performed.

There have been some reports on the drainage of liver lesions, liver cysts without biliary communication, liver abscesses, and bilomas. The long-term efficacy and safety of EUS-guided drainage for simple or infected liver cysts has also been reported.^[4,16]^ However, there have been no reports describing EUS-guided drainage of a liver cyst with biliary communication, such as in our case. In most cases involving EUS-guided drainage of liver lesions, the puncture route was through the stomach, and plastic or metal stents were used. In other cases, nasal drainage tubes were used (Table 1). Adverse events such as segmental cholangitis due to stent placement, bleeding due to injured intervening vessels during tract dilation, and stent migration, which can lead to perforation, stent dysfunction, and leakage of cavity contents, may occur.^[23,33]^ In our case, no serious adverse events occurred. There have been few reports of complications, including death after EUS-guided drainage due to bleeding.^[33]^ Although safe and effective, the procedure is technique sensitive, and should be performed by a skilled endoscopist.

**Table 1:** Previous reports of endoscopic ultrasound-guided drainage for liver lesions.

**Year**	**Author**	**Age (yr)/sex**	**Disease**	**Stent**	**Size**	**Route**	**Complications**	**Technical success**
2005	Seewald et al^[17]^	39/M	Abscess	Naso-biliary stent	110 mm	Transgastric	No	Yes
2008	Shami et al^[18]^	44/M	Biloma	Plastic	37 mm	Transgastric	No	Yes
2008	Shami et al^[18]^	22/F	Biloma	Plastic	80 mm	Transgastric	No	Yes
2008	Shami et al^[18]^	67/M	Biloma	Plastic	40 mm	Transduodenal	No	Yes
2008	Shami et al^[18]^	39/F	Biloma	Plastic	40 mm	Transgastric	No	Yes
2008	Shami et al^[18]^	43/M	Biloma	Plastic	85 mm	Transgastric	No	Yes
2009	Ang et al^[19]^	58/M	Abscess	Plastic	107 mm	Transgastric	No	Yes
2010	Noh et al^[20]^	60/M	Abscess	Plastic	51 mm	Transgastric	No	Yes
2010	Noh et al^[20]^	50/M	Abscess	Plastic	60 mm	Transgastric	No	Yes
2010	Noh et al^[20]^	69/M	Abscess	Plastic + naso-biliary tube	55 mm	Transduodenal	No	Yes
2011	Itoi et al^[21]^	78/M	Abscess	Plastic + naso-biliary tube	70 mm	Transgastric	No	Yes
2013	Medrado et al^[22]^	73/F	Abscess	Metalic	97 mm	Transgastric	Stent migration during the procedure	Yes
2014	Taguchi et al^[4]^	77/M	Incfected cyst	Plastic	N/A	Transgastric	No	Yes
2015	Tonozuka et al^[23]^	71 (53-94)/M:2, F:5	Abscess	Metalic	Mean 70 mm (35.0-181.0)	Transgastric:6, transduodenal:1, Transejejuni; 0	No	(^*^)
2015	Tonozuka et al^[23]^	62 (19-88)/M:5, F1	Infected Biloma	Metalic	Mean 68.5 mm (22.0-83.0)	Transgastric:5, transduodenal:0, Transejejuni; 1	No	Yes
2015	Koizumi et al^[24]^	37/M	Abscess	Naso-biliary tube	N/A	Transgastric	No	Yes
2016	Ulla-Rocha et al^[25]^	76/M	Perihepatic biloma	Plastic	120 mm	Transgastric	No	Yes
2016	Ogura et al^[26]^	66.5 (31-84)/M:4, F:3	Abscess	Metalic	74.6 mm (61.9-99.3)	Transgastric	No	Yes
2016	Kumta et al^[27]^	55/F	Abscess	Metalic	N/A	Transgastric	No	Yes
2017	Yamamoto et al^[28]^	65/M	Abscess	Metalic	N/A	Transduodenal	No	Yes
2020	Cassis et al^[29]^	65/M	Biloma	Metalic	200 mm	Transduodenal	No	Yes
2021	Chandra et al^[30]^	30/M	Abscess	Plastic + naso-biliary tube	N/A	Transgastric	No	Yes
2021	Chandra et al^[30]^	24/M	Abscess	Naso-biliary tube	N/A	Transgastric	No	Yes
2021	Chandra et al^[30]^	55/M	Abscess	Naso-biliary tube	N/A	N/A	No	Yes
2021	Molinario et al^[31]^	82/M	Abscess	Metalic	100 mm	Transgastric	No	Yes
2021	Baron et al^[32]^	80/F	Cyst	Plastic	140 mm	Transduodenal	No	Yes
2022	Lorenzo et al^[33]^	Mean 49 M:8, F:6	Biloma	Plastic	Mean 49 mm	Transgastric:8, transduodenal:6, transjejuni; 0	One bleeding	(^†^)
2022	Our case	70/F	Cyst with biliary communication	Plastic	70 mm	Transgastric	No	Yes

^*^ Two of the 7 patients needed additional drainage.

^†^ One of the 14 patients died due to significant bleeding.

Another reported treatment, involving EUS for liver cysts, is EUS-guided ethanol injection, which is effective and less invasive for simple liver cysts. However, ethanol injection therapy is not recommended for liver cysts with biliary communication.^[9,16,34]^ In our case, sclerotherapy using minocycline was ineffective. Therefore, EUS-guided ethanol injection was not performed. More than 3 years have passed since the end of treatment, with no recurrence or complications.

In conclusion, EUS-guided cyst drainage is an effective, minimally invasive treatment for liver cysts with biliary communication that were unresponsive to conventional treatments, such as percutaneous drainage, sclerotherapy, and endoscopic transpapillary drainage.

## Acknowledgments

This paper is based on the oral presentation at the 113^th^ Japan Gastroenterological Endoscopy Society Kanto Chapter meeting held at Tokyo Medical University Hospital on December 4 and 5, 2021.

## Author contributions

**Conceptualization:** Kento Shionoya, Kazuya Koizumi.

**Data curation:** Kento Shionoya.

**Investigation:** Kento Shionoya, Kazuya Koizumi, Sakue Masuda, Yuma Suno, Jun Kawachi, Karen Kimura, Makomo Makazu, Jun Kubota, Takashi Nishino, Chihiro Sumida, Junichi Tasaki, Chikamasa Ichita, Akiko Sasaki, Hiroki Hadano, Makoto Kako.

**Supervision:** Kazuya Koizumi.

**Writing - original draft:** Kento Shionoya.

**Writing - review & editing:** Kazuya Koizumi.
